# Exploring the use of masks for protection against the effects of wildfire smoke among people with preexisting respiratory conditions

**DOI:** 10.1186/s12889-023-17274-3

**Published:** 2023-11-24

**Authors:** Holly Seale, M Trent, G. B. Marks, S Shah, A. A. Chughtai, C. R. MacIntyre

**Affiliations:** 1 School of Population Health, Faculty of Medicine and Health, University of New South Wales, Level 2, Samuels Building, Sydney, NSW 2052 Australia; 2 The Biosecurity Program, The Kirby Institute, University of New South Wales, Sydney, NSW Australia; 3 School of Clinical Medicine, UNSW Medicine & Health, University of New South Wales, Sydney, NSW Australia; 4 Woolcock Institute of Medical Research, Sydney, NSW Australia; 5 Research and Education Network, Western Sydney Local Health District, Sydney, NSW Australia; 6 Faculty of Medicine and Health, University of Sydney, Sydney, NSW Australia

**Keywords:** Communication, Air pollution, Public health, Wildfire, Respiratory health, Masks

## Abstract

**Background:**

The impact of wildfire smoke is a growing public health issue, especially for those living with preexisting respiratory conditions. Understanding perceptions and behaviors relevant to the use of individual protective strategies, and how these affect the adoption of these strategies, is critical for the development of future communication and support interventions. This study focused on the use of masks by people living in the Australian community with asthma or chronic obstructive pulmonary disease (COPD).

**Methods:**

Semi-structured phone interviews were undertaken with people living in the community aged 18 years and over. Participants lived in a bushfire-prone area and reported having been diagnosed with asthma or COPD.

**Results:**

Twenty interviews were undertaken between July and September 2021. We found that, during wildfire episodes, there was an overwhelming reliance on closing windows and staying inside as a means of mitigating exposure to smoke. There was limited use of masks for this purpose. Even among those who had worn a mask, there was little consideration given to the type of mask or respirator used. Reliance on sensory experiences with smoke was a common prompt to adopting an avoidance behavior. Participants lacked confidence in the information available from air-quality apps and websites, however they were receptive to the idea of using masks in the future.

**Conclusions:**

Whilst COVID-19 has changed the nature of community mask use over the last couple of years, there is no guarantee that this event will influence an individual’s mask behavior during other events like bushfires. Instead, we must create social support processes for early and appropriate mask use, including the use of air quality monitoring.

**Supplementary Information:**

The online version contains supplementary material available at 10.1186/s12889-023-17274-3.

## Introduction

There is the potential for short- and longer-term adverse health impacts following exposure to smoke arising from wildfires (also referred to as bushfires, forest fires, vegetation fires, or brushfires). In this paper, we will use the term bushfire (mainly used in Australia), which refers to a fire that occurs in forest, scrub, or grassland anywhere in the world [1]. During a bushfire, large amounts of air pollutants, including carbon monoxide, nitrous oxides, hydrocarbons, particulate matter, and volatile organic compounds, are released [2, 3]. During bushfire smoke episodes, particulate matter concentrations are usually much higher than urban background concentrations [4].

Many studies have focused on the impacts of bushfires amongst those directly affected by the event [5, 6], but it is now established that bushfire pollutants can cover large areas of land, even hundreds of kilometres away from the actual bushfire [7, 8]. People with asthma, chronic obstructive pulmonary disease, bronchiectasis, and other chronic lung conditions may experience increased symptoms and need for medications, impaired lung function, and increased risk of hospital admission and mortality [9–11]. For example, a systematic review and meta-analysis showed that an increase of 10 µg/m^3^ of landscape fire smoke (LFS) fine particulate matter (PM_2.5_) levels is positively associated with asthma hospitalisations (RR = 1.06, 95% CI: 1.02–1.09) and emergency department visits (RR = 1.07, 95% CI: 1.04–1.09) [12]. Focusing on LFS events in Sydney, Horsley et al. reported that between 2001 and 2013 there were 184 LFS days [13]. They attributed an estimated 197 premature deaths, 436 cardiovascular hospitalisations and 787 respiratory hospitalisations, due to the fire smoke. Even then, they feel that these estimates may have been conservative due to the small number of air pollution monitors that were available. This is a stark contrast to the number of deaths officially attributed to bushfires (77 deaths between 1901 and 2011) [14].

To reduce exposure, it is recommended that people monitor the air quality, avoid vigorous outdoor activity, spend more time indoors and spend time in air-conditioned venues like cinemas, libraries, and shopping centres [15, 16]. Some public health organisations also suggest that wearing a face mask can mitigate exposure to bushfire smoke [15, 16]. However, recently it has been suggested that this general advice, while useful for brief air-pollution episodes, may need to be more detailed for events that last weeks to months. With regards to P2/N95 face masks, while they provide efficient filtration of particles (if well-fitted), they are not tested for general use in children [17]. They do not confer protection from exposure to toxic gases in bushfire smoke (e.g., carbon monoxide, nitrogen oxides, and volatile organic compounds) [9]. It has been recommended that more precise information about the benefits and drawbacks should be provided by health authorities to support community members’ use of these products during short and longer-term events [18]. A qualitative approach was adopted to understand the current reality around mask use and the factors influencing people to (or not to) wear a mask during bushfire events [19]. We were interested in people sharing their experiences to capture a holistic picture of the factors that trigger, support, and impact the use of health protection activities during bushfire episodes.

## Methods

### Study design

Twenty semi-structured phone interviews were undertaken with people living in the community across Australia who self-reported a diagnosis of asthma or COPD. The study was conducted between July and September 2021.

### Ethics approval

The Human Research Ethics Advisory Panel at the University of New South Wales reviewed and approved this study (HC200477). Informed verbal consent was collected from all participants and recorded at the start of the interview. Participants were only included in the study when full verbal consent had been received, and participants were informed they could withdraw at any time. No participants approached for an interview withdrew. There was no established relationship between the researcher and the participant.

### Participants

Participants who were enrolled in a randomized controlled trial (RCT) of mask use for mitigation of adverse respiratory outcomes during the bushfire season were invited to participate in the semi-structured interviews. To be eligible for the RCT, participants must have been 18 years and over, living in a bushfire-prone area (as defined by fire services in NSW, Victoria, ACT, Tasmania, Queensland, Tasmania, Northern Territory, Western Australia, and South Australia); and diagnosed as having asthma or chronic obstructive pulmonary disease (COPD). Among those who completed the first year of the trial, a sample of participants (15 women and 5 men) was purposefully selected based on the data collected during the clinical trial. The aim was to interview a mix of trial participants by gender, age, location in Australia (including urban and semi-urban locations), and randomization group (surgical masks vs. P2 masks vs. avoiding outdoor exposure to smoke, using guidance from the Victoria Government). The interviewer (HS) was unaware of the randomization group at the beginning of the interview. A gift voucher was given to all participants to compensate them for their time.

### Data collection and analysis

An interview guide was developed and reviewed by the researchers (HS, MT) to identify critical areas of interest for the study based a review of the published literature and to support the interpretation of the findings from the clinical trial. The questions related to the following topics: experiences around the use of masks, barriers to use of masks, and participants’ thoughts on what needed to be done to enhance adoption of masks during bushfires. Questions were asked in an open-ended manner to allow room for expansion and longer conversation, in order to gather deeper and more comprehensive insights [20]. All caution was taken to ensure that the wording of the questions did not affect the outcome of the interview or impose answers. The topic guide served only as a general direction for the researcher during each interview. Paraphrasing and additional questions were added to seek clarification. Interviews lasted approximately 30–40 min and no repeat interviews were undertaken. Data collection continued until the lead researcher was satisfied with *data sufficiency*, or the richness of the data that was generated from the interviews would lead to rigorous data analysis [21]. Unlike the concept of data saturation, which posits that further data collection will not yield additional valuable insights [22], the idea of data sufficiency acknowledges that within a research paradigm acknowledging the uniqueness of human experiences and the socially constructed nature of data, researchers can continually delve into a reservoir of new insights by iteratively refining interview guides, sampling new participants, and conducting multiple rounds of data generation and analysis. The recurrence of concepts and themes across the dataset indicated that the sufficiency threshold was likely met, if not exceeded, for this study’s specific dataset, if not the saturation threshold.

Our data analysis adhered to a comprehensive six-step thematic analysis framework developed by Braun and Clarke, which ensured a rigorous process leading to analytical sufficiency, where the research team gained confidence that the collective analysis effectively encompassed the insights provided by key informants [23]. Data analysis was conducted throughout the data collection processes, and following the main analysis undertaken after its completion. Areas that suggested further exploration in subsequent interviews were also identified. Using an inductive approach to the data analysis, HS started to develop codes that were linked to the data themselves. HS has a background in trials and other studies relating to the use of masks, but this has previously been limited to healthcare settings or infectious diseases outbreaks. This process would then facilitate data coding without using an existing theoretical or coding framework, or referring to our own presumptions about what the analysis would show. Subsequently, a second investigator (MT) coded a proportion of the transcripts, iteratively refining the scheme to encompass emerging themes arising from the interview responses. To facilitate coding, data categorization, and analysis across various participant perspectives, we employed qualitative data analysis software, specifically QSR International’s NVivo 12. The research team convened to discuss and reconcile their respective coding categories, addressing any discrepancies through thoughtful dialogue and achieving consensus through negotiation. The process of data analysis and interpretation was an iterative one, involving active participation from all team members, with the aim of identifying and mutually agreeing upon emergent themes and assessing their face validity. By listening carefully to the interview audios and verifying the data during transcribing, we attempted to ensure the study’s dependability. An overview of the research team, study design and analysis is described in Appendix A using the COREQ reporting format [24].

## Results

The themes identified from analysis of the interview data are described in detail below.

### Limited past experiences with masks for bushfires

Participants reported very little use of face masks for protection against the adverse effects of smoke from bushfires. Most referred to using masks for COVID-19, gardening, painting, or cleaning/dusting, or for occupational reasons, such as working in industry or healthcare. Of those who did speak about using masks, they often related the use to a particular bushfire event (i.e., the 2019 fires that impacted the Australian states of NSW and Victoria) that triggered them to start using the products (or something to cover their mouths like a handkerchief). However, there were others who related their experiences of being evacuated during a bushfire event and recalling people wearing masks.

*“We were evacuated in 2015… I think. I don’t think we had a mask, other than just tying my scarf around my neck– around my face. We were caught up in a sense in that fiasco. Other than that I really honestly can’t remember” (Participant 3)*.

*“I had begun to use masks during the 2019 bushfire season, as I found them to prevent some of the smoke getting into my lungs and triggering my asthma. It was more a trial and error thing to see if it would work. I didn’t have access to many masks at that stage. I had been struggling through that 2019 season. I just was trying to find a way to stop that feeling of the smoke getting in”. (Participant 8)*

*“Everybody was in the same boat. We were all hacking all over one another. It’s a terrible sound, isn’t it? There were no masks, nobody had masks. Nobody thought about masks. There may have been the odd few that may have had a mask if I remember back and think about it, but as a rule, no”. (Participant 13)*

Amongst those who were using masks, reference was often made to going to the local hardware store to purchase “*whatever was available”*. Generally, this referred to surgical or P2/N95 face masks that they were able to source.

*“until we got the fires here in 2019. Up till then, I just randomly chose a mask at [hardware store] or something like that, not really knowing anything about it, and not being sensible enough to ask anybody that knew anything about it” (Participant 9).*

Despite not using them previously, most participants acknowledged that they would consider using a mask in the future during times of bushfires. They acknowledged that the masks were not all equal and that the N95/P2 masks were superior to the other products available. When comparing the different masks available, one participant spoke about the ability to smell the smoke in one mask (surgical) and not in another (N95/P2). The N95/P2 were also preferred as they were easier to breath in and felt less claustrophobic.

*“No, they’re definitely not equal. The cloth masks aren’t doing anything, really. The ash not getting into your mouth is the only thing the cloth will stop. You can still smell the smoke through the cloth”. (Participant 8)*

### My favorite strategy is avoidance

While participants spoke about low levels of awareness and use when it comes to using masks for bushfires, a larger number spoke (unprompted) about a range of other strategies that they have used in the past during smoky conditions. The key approach adopted was attempting to avoid the bushfire smoke. Participants also referred to stuffing window and door frames with towels, using air-purifiers and air-conditioners, or just keeping the house “shut up”.

“*I didn’t go out anywhere. I sat home, and tried to keep the house locked up as much as I could, but I’ve a dog that was in and out every five minutes. I couldn’t really keep the smoke out*.” (Participant 20).

*“When the fires were getting bad here the last time, no, 2013 fires that was pretty bad, what I did was I just wet towels and put them around all the doors so that the smoke couldn’t come in through the doorway”. (Participant 4)*

*“First of all, I shut the house up. Then I stuffed hand towels into the windows to close the air gaps as much as I could, and I just bought an air purifier. I had that going 24/7, and that was just good enough to keep me going at home. Most of the time I bunkered down at home. If I did have to go out, I had to plan it because I couldn’t even take one breath out. Because as soon as I tried to breathe, with all the smoke particles, all the dust, I’d instantly have an asthma attack”. (Participant 5)*

*“It’s got to be better than nothing. I suppose, after having had to wear a mask for COVID, even if just for a short time, it’s got to be better than nothing. I couldn’t say it’s not going to reduce my asthma in impact of asthma….It would be better if I could be outside for a little bit longer… I still think avoiding it is probably the better option, but if you’ve got to be out and about, I think a mask has got to be better than no mask” (Participant 7)*.

When they had to go out, participants spoke about carefully planning their trips so that they could avoid having to walk around. They would park their cars as close as possible to their destination and hold their breath until they were back inside. Others avoided going out or modified their practices so that they would not need to go out, and so relied on home delivery to receive their food shopping.

*“I’d walk away into another room if someone wanted to open the door. Again, not that we were doing that because the smoke was bad on the days that was bad. We were protecting everybody. I just remember them saying to me, “Look, it’s a bad day; you’re not going anywhere.“ (Participant 8)*.

### Relying on visual triggers for use

Participants spoke about relying on their sense of sight and smell when it came to trying to work out whether they needed to be aware of the air quality. They spoke about looking at the sky and trying to work out the visibility level.

*“I’d look at the color of the sky, because it might look clear ground level but if it was still hazy in the sky then it wasn’t a priority to go out unprotected”. (Participant 5)*

The use of air-quality apps was mentioned by participants as another mechanism to assist with checking whether they should go out. However, there were very mixed views about the usefulness. Participants spoke about errors with the systems, about them not being user-friendly and not trusting the information. For those who did not trust the information, the issues appeared to stem from the fact that the data-collection point was too far away from where they lived for the information to be reliable.

*“Sometimes they lag behind the stuff I get on the email from the department of environment. There might be a little bit of a time lag getting it through”. (Participant 1)*

*“Some data will say that air quality is inferior. I did a bit of research myself to find out how they’re measuring it, that sort of stuff. Because I’d often be on days where the air was really clean, I had no asthma. Now saying it was the high ratings of P2 and whatever they’re called. I’ve realized that where I am taking those readings either from the south or more the city area” (Participant 6).*

Participants spoke about firstly using visual cues to help with judging the air quality. However, in many instances they would then use the air-quality apps or websites to confirm or double check the air quality ratings. They might check the information before leaving the house or on days when they are outside for sport. Amongst the limited number of participants who regularly used the app, they spoke about checking them every morning and about getting regular alerts via their phone or watch. A couple of the participants spoke about relying on information from websites (from the Department of Environment) and from the apps. To help build trust in the systems, participants felt it would be helpful to know more about where the information is being sourced from (i.e., the location of the monitoring stations).

“*Every morning I get the air quality. I don’t know how accurate they are, I’ll be absolutely honest, but I get the air quality come through on my watch. It has once suddenly pinged me later in the day. There was a warning that there was a bit of an air quality issue…I trust it because it’s the Department of Environment”. (Participant 9)*

### We need a kick-start to get people to use masks

Very few people mentioned that they had received any advice or reminders from their General Practitioners (GPs) or a healthcare professional to wear a mask during times when there is bushfire smoke around. Getting a recommendation from a healthcare professional was suggested to encourage more people, especially those who are immune compromised, to adopt bushfire precautions.

*“My doctor believes that I should wear a mask anytime that the air quality is poor. He reminds me of this every time I see him pretty much…. I generally ignore him, though.” (Participant 15)*.

“*I did see a GP at that time (during the bushfires) because I had to get prednisone…. The advice again was to stay indoors. Keep the windows closed. Run the air conditioning and just avoid being outdoors basically. The GP didn’t say wear a mask or any of that. They asked me more around what my activity levels are were, when am I going outside and why. If those things are avoidable, don’t do them*”. (Participant 7)

To support the adoption of masks, it was suggested that information could be sent out via websites/social media accounts of groups such as Asthma Australia, the Lung Foundation etc. Receiving an SMS alert was also nominated as a useful strategy to not only let people know about air quality levels, as well as nudge people that they should consider adopting a behaviour which is appropriate to the air quality level.

*“I don’t watch a lot of TV. I’m very selective in my television viewing. I still think your best thing would be an app. I’ve got an app for Fires Near Me, because we’re also in a bushfire zone as well where we live, our streets”. (Participant 17)*

“*when they’re bushfires in those areas, you could get a notification saying there’s a bushfire or some backburning or something, or even bad smoke, like a day whether it’s just bad smoke, for example. That would be great if you could get notified like that*”. (Participant 11).

Other suggestions including getting reminders/prompts to apply the bushfire precautions via Royal Fire services, local radio stations, via local council/government notices, via the news or on social media such as local community networks or groups focused on the health conditions.

*So, I think like having it normalized in the media, so, if it’s smokey day and there’s somebody giving a talk on what’s happening, like seeing it on the TV, seeing it in articles. I think people that have to work, I think should really be empowered and encouraged to wear masks (Participant 16)*.

*“There’s no point putting anything in the local paper because nobody buys the local paper. It’s probably going to die off. Maybe a council, notice. Because that’s one thing that people do look at council notices” (Participant 20)*.

## Discussion

The low levels of mask use captured during these interviews are reinforced by the results from an earlier study we undertook to explore mask use for bushfires in Australia [25]. Community members with and without respiratory conditions were surveyed to compare health effects of the 2019/2020 bushfires (a period of unprecedented bushfires affecting multiple states resulting in 400 excess deaths and 3000 additional hospitalisations). Respondents 18 years or over were recruited from bushfire-prone area (as defined by fire services in NSW, Victoria, ACT, Tasmania, Queensland, Tasmania, Northern Territory, Western Australia, and South Australia). The survey captured self-reported use of surgical or P2 masks during the bushfire time. Self-reported mask use during the bushfire time was limited; 20% of the respondents with respiratory conditions reported using a mask or respirator during the period of bushfire, dropping to 9.7% for those without a respiratory condition. This was despite a high proportion of both cohorts of respondents reporting exposure to bushfire smoke in the 12 months prior (70% of the cohort with respiratory conditions vs. 63.9% without respiratory conditions.

Internationally, few studies have been conducted looking at the use of masks during bushfire events [26]. Most studies have been conducted in the United States following the bushfires in California. One such study exploring the health impacts of wildfire exposure on pregnant women found that most (80%) reported wearing masks (most (84%) were N95/P2) [27]. An older US study, conducted during the fifth largest US wildfire in 1999, reported a high number of people wearing non-filtered masks or bandana’s, limited use of masks by people evacuated from their homes, and a positive association between mask use and outdoor exposure [28]. Regarding the issue of mask use and increased participation in outdoor activities, concerns have also been raised that masks may give the wearer a false sense of security (or self-efficacy) and may result in their continuing to participate in outdoor activities or potentially lowering their adoption of other strategies during periods of smoke [29].

Risk mitigation strategies such as mask wearing, staying indoors, and using air-filters are closely linked with how community members perceive their vulnerability and the threat of bushfires or the smoky environment, their level of understanding about the strategies, whether they can access/afford the products, and whether they perceive the strategies to be effective. Obviously, social norms also play a role in promoting people to take up a protective health action, like mask wearing, as evident during the COVID-19 pandemic [18, 30]. Studies focused on the environment or natural hazards have found that perceptions of subjective norms are associated with individual mitigation actions. For example, a Chinese study focused on air pollution identified that both descriptive and injunctive social norms predict mask use [31].

The use of their senses as an alert system to smoke was a common feature amongst our participants. This approach has also been reported elsewhere, with one study finding that amongst their participants there were those who relied exclusively on their senses (smell and sight) and did not search for any information about bushfire smoke. The connection between smelling the smoke and believing that they will experience a negative health outcome was common. The role and influence of sensory experiences on perceptions of threat has been previously noted in other studies on air pollution [32], with the suggestion that these perceptions can influence future smoke perceptions and behaviours. In comparison to relying on their senses, the reliance on air-quality apps was mixed amongst our participants. Whereas other studies have reported that community members constantly check information and use the reports to help decide on their activities for the day (described as a coping strategy) [30], amongst those we interviewed there were feelings of mistrust towards the systems. Concerns that the information from the systems was inaccurate given the location of the sensors, or not up to date, certainly influenced people’s use of the air-quality apps and reports. In some cases, participants acknowledged that they did not have a good understanding of how the data was collected and this may have influenced their feelings. From these results, clearer information provided to support people’s understanding about the role of these air-quality apps, the limitations associated with them, and instructions on how to maximise the usefulness, could benefit in supporting trust and future use. It may also be useful to relate how people’s senses and what they are already contextualising in terms of seeing/smelling and tasting (environmental cues), relate the information being provided by the air-quality apps. Of note, alternatives should be made available in rural and remote locations where there is limited ground-based air quality monitoring.

Amongst our participants there were mixed views regarding the more appropriate ways to communicate messages around mask use and other precautions prior to and during periods of bushfires. Focusing firstly on supporting awareness and understanding of people with respiratory conditions about the need to protect themselves during bushfires and the most appropriate strategies to use, there was a strong recommendation to work with not-for-profit organisations whose mission is to support those living with the focus health conditions. Asthma and Lung Foundations were singled out as being the gateway into these communities at risk. Internationally the American Lung Association has included information about wildfires on its websites, with recommendations that focus on before, during and after wildfire events [33]. Looking at the information related to mask use on the website, the Association recommendations focused on educating people to carefully consider the type of mask being used, highlighting that ordinary dust masks and cloth facial coverings will not help. They also caution people to consider consulting with their doctor before using a N95/P2 mask. Similar recommendations are given by the Asthma and Allergy Foundation of America [34]. The Foundation provides a strong recommendation that if people must spend time outside during bushfires that they wear a N95/P2-rated mask. Importantly they also include a visual breakdown of the Air Quality Index and what the different categories mean. To enhance the information provided by these organisations, consideration could be given to ensuring people understand the rational for needing to wear a N95/P2 mask, as well as how to fit and reuse the product.

During an emergency, it is critical that clear messages must be given about the threat and the precautions that should be adopted. The use of SMS and other app-based reminders was recommended by our participants, as well as ensuring that messages go out via local radio and TV channels. The challenge is ensuring that the messages are understood. A study conducted following the bushfires of 2007 in San Diego, California found that while most people (n = 1802 residents living within the county) could recall hearing a fire-related health message, very few could recall hearing technical messages regarding the use of N95/P2 masks and few (> 10%) followed those specific recommendations [35]. So that while nontechnical message recall was great, the nuances around mask use was lost. The authors suggested that to improve the clarity in the communication of risk and the recommended precautions, that videos, personal testimonies, and easy-to-read materials should be adopted. Based on the lessons learnt during the COVID-19 pandemic, the use of social media and private messaging services should also be considered [36]. Consideration could be given to pre-enrolling community members in auto-alert systems so that they can receive an automated call (in their pre-determined) language about the situation.

It is important to remember that public health messages alone will probably not elicit behaviour [37] and so it is critical that efforts are made to also promote social support and communication between peers, family and friends. Encouraging people to share information about air quality, how to interpret the threat and the need for protective action may improve the person’s perception of the threat, as well as their behaviours (response efficacy) during a crisis [38]. The act of giving someone a mask to wear may influence the subjective norm and nudge the person to wear a mask [30]. Further work is needed to look at how social support is given before, during and after a bushfire event amongst communities directly impacted by the fire, as well as those communities impacted by the bushfire pollutants. Beyond looking at the role of community messaging and communication efforts, future research could also focus on the role that healthcare providers have on advocating and promoting uptake of different preventive strategies for bushfire smoke exposure. Based on the limited number of prompts that participants could recall receiving from a healthcare provider, there may be a need for further work in supporting the understanding of primary care providers.

Biases require particular attention in the interpretation of these findings. Participants in this study were already enrolled in a large RCT focused on the effects of surgical and P2/N95 masks for bushfires. They would have received information as part of the consenting process about the aims of the larger study and the rational for undertaking the work. This information may have influenced their responses during the interviews. In addition, the interviews were undertaken during a low-fire period and during a surge in COVID-19 cases (requiring residents in some areas to wear a mask when out in public). Their responses to the questions may have been influenced by what was happening during COVID and their feelings towards the use of masks for infection control purposes. For some participants, it had been over a year since the large bushfire events and so the results would be subject to recall and selection biases. Lastly, participants were mainly located in urban or semi urban areas, with limited representation from remote community settings. Further demographic information was not available for the participants.

Moving forward, we recommended that clearer guidance be given around the rationale for mask use, as well as the instructions of what mask to use and when it should be used during bushfire events. Key learnings from COVID-19 and other natural disaster emergencies around how to maximise communication efforts must be considered for future smoke events to ensure equity in the delivery of information. Further research is needed around how to assist people to engage with air quality websites and apps so they can mitigate risk earlier, the interaction between this information and environmental cues (especially in times of conflict or inconsistencies), and how to relate this information to the adoption of mitigation strategies.

## Conclusion

Whilst the COVID-19 pandemic has changed the nature of community mask use over the last couple of years, there is no evidence that this event will influence an individual’s mask behavior during other events like bushfires. To ensure protection against harmful smoke exposure, we must create social support processes for early and appropriate mask use, including the use of air quality monitoring. This study has demonstrated the need for increased clarity and further development of communication during bushfire events, ideally around the rationale for mask use, the specific type of mask and instructions of when they should be used.

### Electronic supplementary material

Below is the link to the electronic supplementary material.

Supplementary Material 1

Supplementary Material 2

## Data Availability

The datasets used and analyzed during the current study are available from the corresponding author on reasonable request.

## List of abbreviations

COPD: Chronic obstructive pulmonary disease
GPs: General Practitioners
LFS: Landscape fire smoke
RCT: Randomized control trial
