# Supplementary material for: Exploring the use of masks for protection against the effects of wildfire smoke among people with preexisting respiratory conditions
Source: BMC Public Health. 2023 Nov 24;23:2330. doi: 10.1186/s12889-023-17274-3 (PMC10668508; doi:10.1186/s12889-023-17274-3)
Supplement: Supplementary file 1 — Supplementary Material 1 [file 12889_2023_17274_MOESM1_ESM.docx]

**CORE-Q Consolidated Criteria for Reporting Qualitative Research**

Based on Tong, A., Sainsbury, P., & Craig, J. (2007). Consolidated Criteria for Reporting Qualitative Research (COREQ): A 32-item checklist for interviews and focus groups. *International Journal for Quality in Health Care*, *19*(6), 349–357.

| **No** | **Item** | | **Description** |
| --- | --- | --- | --- |
| **Domain 1: Research Team and Reflexivity** | | | |
|  | **Personal Characteristics** | | |
| 1 | Interviewer | HS | |
| 2 | Credentials | The interviewer has Bachelor, Master and PhD qualifications in social research, and public health | |
| 3 | Occupation | Academic staff (HS) | |
| 4 | Gender identity | Female | |
| 5 | Experience and Training | Experienced researcher and interviewer, has undertaken several qualitative studies, trained in interviewing and qualitative analysis. | |
|  | **Relationship with participants** | | |
| 6 | Relationship established | None of the interviewees was known to the interviewer before the interviews. | |
| 7 | Participant knowledge of the interviewer | The interviewer were not known to participants before the interviews were conducted. | |
| 8 | Interviewer characteristics | Established researchers with experience in public health and respiratory protection. | |
| **Domain 2: Study Design** | | | |
|  | **Theoretical Framework** | | |
| 9 | Methodological orientation and theory | Exploratory | |
|  | **Participant Selection** | | |
| 10 | Sampling | Participants who were enrolled in a randomized controlled trial (RCT) of mask use for mitigation of adverse respiratory outcomes during the bushfire season were invited to participate in the in-depth interviews. | |
| 11 | Method of approach | Emails that included information on the study, participant information and consent forms. | |
| 12 | Sample size | 20 | |
| 13 | Non-participation | Reason for non-participation was not documented due to recruitment approach. | |
|  | **Setting** | | |
| 14 | Setting of data collection | Conducted via online videoconference platform and telephone | |
| 15 | Presence of non-participants | No non-participants were present for the interviews | |
| 16 | Description of sample | Participants must have been 18 years and over, living in a bushfire-prone area (as defined by fire services in NSW, Victoria, ACT, Tasmania, Queensland, Tasmania, Northern Territory, Western Australia, and South Australia); and diagnosed as having asthma or chronic obstructive pulmonary disease (COPD). | |
|  | **Data Collection** | | |
| 17 | Interview guide | Provided in advance upon request | |
| 18 | Repeat interviews | No | |
| 19 | Audio/visual recording | Audio recording | |
| 20 | Field notes | Limited reflective notes were captured following interviews. These notes were not included in the qualitative analysis. | |
| 21 | Duration | Ranged between 20 minutes to 40 minutes | |
| 22 | Data saturation | Sampling continued past basic conceptual/thematic saturation to ensure a range of perspectives were included. | |
| 23 | Transcripts returned | No | |
| **Domain 3: Analysis and Findings** | | | |
|  | **Data Analysis** | | |
| 24 | Number of data coders | Initial coding was done by HS. Codes, concepts and higher-order themes were discussed by all authors. | |
| 25 | Description of the coding tree | No, not necessary given the focus of the analysis is largely descriptive rather than being theory-driven. | |
| 26 | Derivation of themes | Codes and concepts were derived through open coding of interview transcripts, these were then tested and consolidated into higher order themes. | |
| 27 | Software | NVivo | |
| 28 | Participant checking | No | |
|  | **Reporting** | | |
| 29 | Quotations presented | Yes, selectively to illustrate findings | |
| 30 | Data and findings consistent | Yes | |
| 31 | Clarity of major themes | Yes | |
| 32 | Clarity of minor themes | No, the focus in this paper is on major analytic findings and higher-order themes, given the focus of the study. | |
