# Supplementary material for: Exploring the use of masks for protection against the effects of wildfire smoke among people with preexisting respiratory conditions
Source: BMC Public Health. 2023 Nov 24;23:2330. doi: 10.1186/s12889-023-17274-3 (PMC10668508; doi:10.1186/s12889-023-17274-3)
Supplement: Supplementary file 2 — Supplementary Material 2 [file 12889_2023_17274_MOESM2_ESM.docx]

Can you tell me about any previous experiences with using masks or respirators?

Are you aware of any recommendations around the use of masks/respirators for bushfire smoke? Have you previously received any communication about using masks or respirators when bushfire smoke is present?

How important do you think masks and respirators are in reducing health issues from smoke? (Prompts: How effective are the mask and respirators? Do you feel safe when you use the mask/ respirators?) How do they compare to using other strategies? (Prompt ask about other strategies)

Can you tell me about your experiences with using [insert mask or respirators] during the trial (Prompts: issues/challenges, prompts to use etc)

Do you think there are differences in amount of protection given to you by cloth a mask, a surgical mask or N95 mask?

Looking forward, would you use these products in the future? What would support you to use it? Would you encourage others to do it?
